# Exploring the potential of virtual reality in nursing education: learner’s insights and future directions

**DOI:** 10.1186/s41077-025-00337-3

**Published:** 2025-03-05

**Authors:** Frode Johansen, Helge Toft, Odd Rune Stalheim, Maria Løvsletten

**Affiliations:** 1 Faculty of Social and Health Sciences, Terningen Arena, Elverum, Hamarveien 112 2406 Norway; 2 Faculty of Education, Midtbyen Park, Skoleveien 4, Hamar, 2318 Norway; 3 Department of Educational Studies in Teacher Education, Hamar, Norway; 4 Section for Nursing, Elverum, Norway; 5 Section for Mental Health and Rehabilitation , Elverum, Norway; 6 University of Inland Norway, Elverum, Norway; 7 Innlandet Hospital Trust, Brumunddal, Norway

**Keywords:** Virtual reality, Nursing education, Pedagogical integration, Learner-centred design, Clinical simulation

## Abstract

**Aim/objective:**

This study aims to explore the perceptions of nursing students on virtual reality (VR) technology, focusing on its utility, relevance, user-friendliness, and potential for broader integration into nursing education and other educational domains.

**Background:**

VR is increasingly utilized in education, providing immersive and interactive learning experiences. Despite its potential, there are concerns regarding its practical application and alignment with educational objectives across various disciplines.

**Design:**

The study employed an exploratory descriptive design using focus group interviews to gather qualitative data from nursing students.

**Methods:**

Semi-structured interviews were conducted with 15 nursing students across three focus groups. The discussions centered on their experiences with VR in medication management training, focusing on perceived utility, user-friendliness, and motivation for future use. Data were analyzed using thematic analysis to identify key themes and insights relevant to educational technology adoption.

**Results:**

Students acknowledged VR’s potential for providing a safe and enhanced learning environment. They appreciated the opportunity to practice without real-world consequences but expressed concerns about technical complexity, lack of user-friendliness, and the absence of realistic clinical scenarios. The need for better alignment of VR content with actual educational needs and more user-friendly interfaces was emphasized.

**Conclusion:**

The findings highlight the importance of aligning VR technology with the specific needs and learning objectives of students in various educational settings. Continuous dialogue with end-users is crucial for enhancing the educational effectiveness of VR. This study provides valuable insights for educators and developers to refine VR applications, contributing to the development of solutions that meet practical and educational requirements across different disciplines.

## Background

There is a pressing need to critically evaluate how VR is being integrated into nursing curricula, focusing on whether these technologies genuinely enhance learning or merely add a layer of technological engagement without substantive pedagogical value [1]. Critical scrutiny should also extend to the cost-effectiveness of VR implementations, examining whether the benefits justify the investments in VR technology [2]. Such an approach would ensure that VR supports nursing students in acquiring theoretical knowledge and also effectively translates these skills into practical, clinical proficiency [3].

Adopting virtual reality (VR) technology in educational paradigms marks a pivotal shift towards innovative teaching and learning methods, particularly within disciplines like nursing that stand to gain from immersive learning experiences [4, 5]. As VR becomes more embedded in educational contexts, its potential to supplement traditional learning methods and enhance student engagement is increasingly recognized [6, 7]. Our qualitative investigation centers on the perceptions and reflections of nursing students regarding the utilization of VR technology and virtual medication management training, enriching our understanding of the subjective dimensions [8] influencing the adoption and effectiveness of VR in educational settings.

This study aims to fill critical gaps by examining how well VR educational tools align with the academic needs and learning styles of nursing students. By investigating nursing students’ experiences with VR, the study will highlight its impact on learning, identify integration challenges, and explore motivational factors. It will also provide practical recommendations for educators and policymakers to enhance VR applications, contributing to theoretical discussions on educational technology and optimizing curriculum development for better outcomes. Through theories such as the technology acceptance model (TAM) [9], this research aims to achieve insights that can enhance our understanding of how individuals engage with new technology, which in turn can have significant implications for the delivery of nursing education.

The integration of VR into nursing education has primarily been driven by its capacity to simulate medical environments and procedures, providing a risk-free platform for students to enhance their clinical skills and decision-making capabilities [10, 11]. While some studies highlight VR’s efficacy in enhancing theoretical knowledge, with meta-analyses showing improvements in knowledge retention compared to traditional learning methods [12], the evidence remains mixed regarding improving practical skills, satisfaction, and performance efficiency. This variability suggests a gap in VR’s application effectiveness across different learning environments and setups [13, 14].

Current research predominantly focuses on the immediate benefits of VR, such as enhancing engagement and providing interactive learning experiences [15, 16] but often overlooks the deeper pedagogical integration necessary for sustained skill retention and application in real-world clinical settings [17]. The diversity in VR training modules’ design often lacks a standardized approach, affecting the consistency and comparability of research findings [18]. To address this, there is an increasing emphasis on providing comprehensive descriptive details of VR interventions, including hardware specifications, task design, and user interactions. Such descriptions are critical to ensure repeatability across studies and to facilitate cross-design analyses, which are essential for identifying best practices and informing future VR developments in educational research [19].

The technology acceptance model (TAM), introduced by Davis in 1985 [20], offers a framework for understanding how users come to accept and use technology. According to TAM, perceived usefulness and perceived ease of use are primary factors influencing an individual’s intention to use a technology [9, 21]. This model has been further validated and extended in subsequent research [22, 23]. In the context of VR in nursing education, TAM can help explain the participants’ motivation based on their experiences of the technology’s utility, relevance, and user-friendliness, which are crucial for its effective adoption and integration.

## Methods

### Research design

This research employed an exploratory descriptive design, utilizing focus groups for data collection [24]. The study was abductive in nature; it commenced with a theory-driven approach with semi-structured interview guides designed to explore specific theoretical constructs. This theory-driven approach is inherently deductive. Concurrently, it embraced an inductive analysis, allowing for the emergence of new themes and insights directly from the data [25].

### Setting and participants

The study engaged 15 participants, distributed across three focus groups, and were in their first and second years of study (Table 1). An equitable representation of gender and age among the participants was observed, a balance that occurred naturally rather than by intentional selection. For the recruitment process, instructors at the nursing education program were used to inform and recruit students to participate in the focus group interviews. Students directly contacted us to express their interest, thus being included in the study. The focus group interviews aimed to discuss their experiences with the trial of VR technology and software for immersive virtual medication management, which they had participated in approximately 3 months earlier. In this trial, participants received a concise presentation of the research project, along with details of the specific investigation, including the research question and objectives. An instructional video with screen captures demonstrated essential tasks and functionalities. Participants could ask questions before starting the VR testing. Five students at a time, each equipped with their own HMD and computer, completed a task in the Virtual Medicine Room.

**Table 1:** Distribution of focus groups by the number of participants and their academic year

Focus group	Number of participants	Academic year
1	4	Second year
2	3	Second year
3	8	First year

**Fig. 1:**
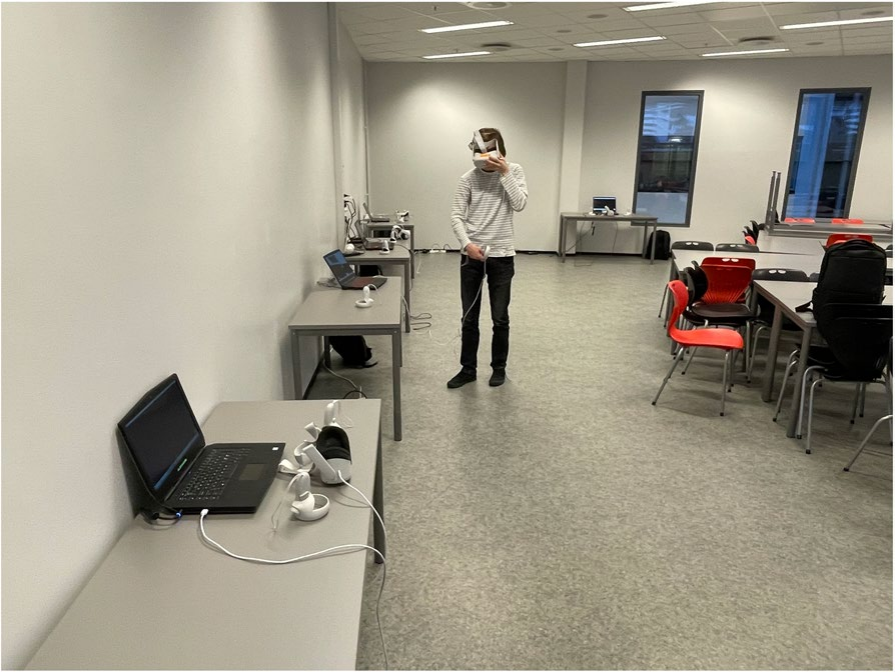
VR station setup for student testing

**Fig. 2:**
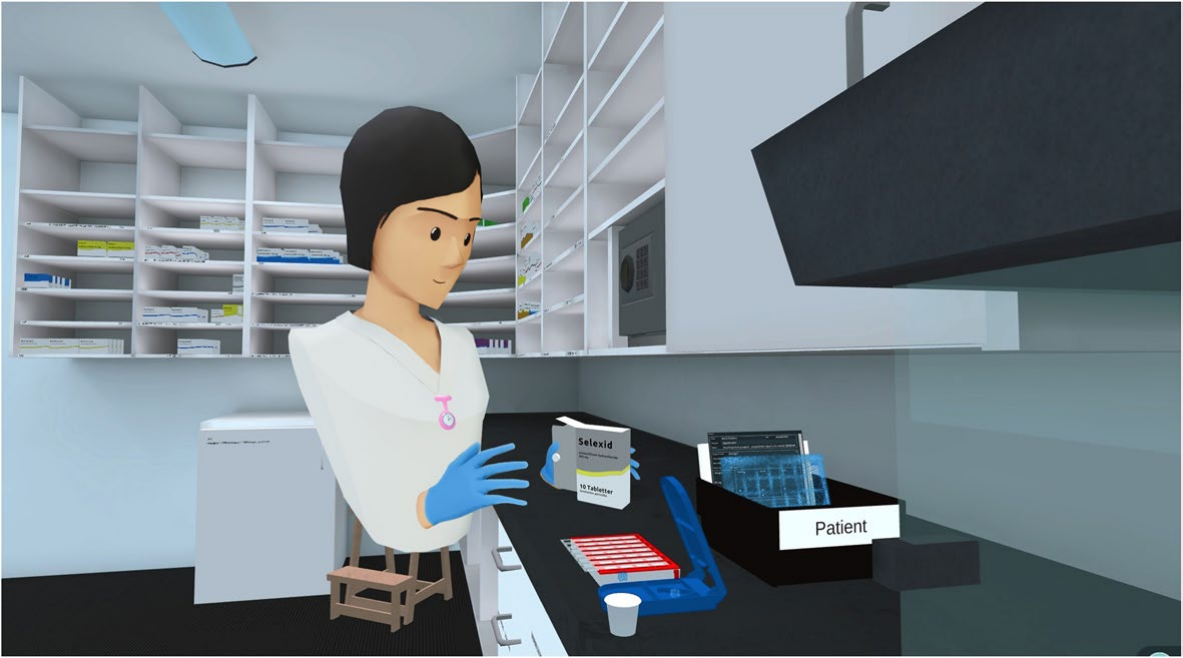
Screenshot of the Virtual Medicine Room

The Virtual Medicine Room was tested using Meta Quest 2 head-mounted displays (HMDs), tethered to PCs for enhanced processing power and stability. Participants navigated the environment using handheld controllers to perform medication management tasks, such as locating the correct medication and placing the appropriate dose into a dispenser. The VR design was minimalistic, with no auditory feedback and limited visual cues, aside from a subdued blue light highlighting key interactable elements like the dispenser’s return location. Each participant engaged individually in a 15-min session, focused on familiarizing themselves with the technology rather than completing predefined goals. Although this trial was conducted as a single-player experience, the software also supports multi-user collaboration (Figs. 1 and 2).

### Data collection

In the data collection phase of the study, participants in the focus group interviews were invited to share their experiences and reflections related to their acquired experiences with VR technology within an educational context. Two researchers were present during these sessions: one led the discussion, while the other observed and took occasional notes. Audio recordings were captured using a Dictaphone app designed to store recordings directly on an approved and secure server, ensuring no local storage occurred. The focus group interview followed a semi-structured interview guide, centering on themes such as the perceived utility of VR, user-friendliness, and the participants’ desire or motivation to incorporate this technology into their education in the long term.

The session concluded with the observing researcher summarizing the interview from their perspective. This summary aimed to encapsulate the discussion’s essence as witnessed by the observer, allowing participants to offer additional reflections and considerations that might not have been touched upon during the interview but were deemed central to the study. This approach facilitated a thorough exploration of the participants’ experiences and insights, contributing valuable data for analysis. The three focus group interviews ranged from 40 to 55 min.

### Data analysis

**Fig. 3:**
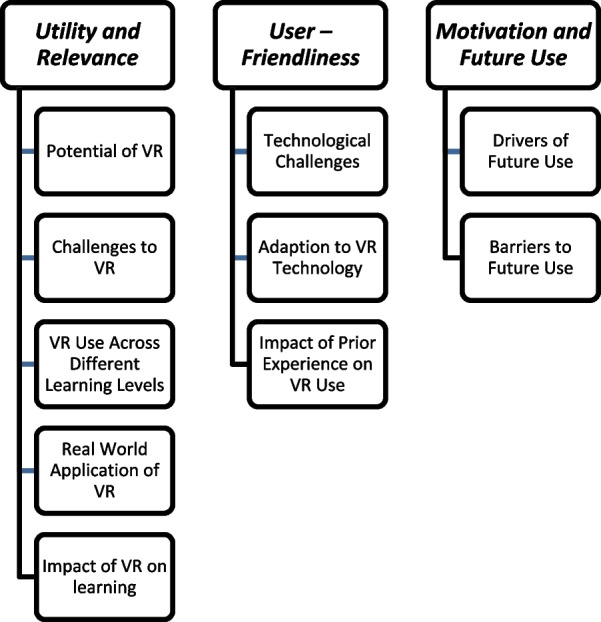
Themes and sub-themes

In the data analysis phase, our methodology was guided by thematic analysis [26]. This analysis adhered to the six steps of thematic analysis proposed by Braun and Clarke (2022), providing a structured and comprehensive framework for interpreting the data collected from the focus group interviews. The research team engaged in a collaborative process that involved individual work, group sessions, and discussions to identify codes, themes, and sub-themes (Fig. 3).

### Ethical considerations

Participants were informed verbally and in writing about their participation in the focus group interview and signed a consent form before participating. The study received approval from the University’s Ethical Committee, ensuring that all ethical standards and guidelines were strictly adhered to (18–2023 Archive: 2023/05337).

## Results

In exploring the integration of VR into educational settings, our study sought to investigate how nursing students perceive and assess the use of VR technology in their education, and how these insights can be leveraged by educators and policymakers to advance and innovate nursing education.

### Utility and relevance

Several participants appreciate VR for allowing practice without real-world consequences, with one noting, “it’s very useful for those in a beginning phase.” The risk-free environment offered by VR is seen as an asset for learning and making mistakes. Another participant metaphorically adds, “It’s a fine seedling here, but we need a flower, so to speak,” highlighting the potential yet calling for further development. Another envisions VR as a “supplement to skill training,” reinforcing its capacity to enhance traditional educational methods.

A common challenge noted across all groups was the technical complexity and learning curve associated with VR technology. A participant pointed out the technical hurdles, saying, “it can become too technical, and [students] have to familiarize themselves with it first,” a sentiment echoed by another who critiques the time investment in learning VR as potentially wasted. Furthermore, another participant's criticism of VR's prioritization over traditional methods, “I don't think this is something the school should spend time on”, reflects a broader concern about resource allocation.

One participant highlights VR's immersive feel: “When I put on the glasses, it felt like I was actually in the medication room at a hospital,” showcasing its potential to simulate clinical environments effectively. However, another pointed out discrepancies between VR and actual medical settings, “What I encountered in VR didn't quite match the reality…it's not quite the same,” emphasizing the challenge of capturing the full complexity of real-life practices. Another called for VR scenarios that mimic the “controlled chaos” of medical rooms, arguing for a more realistic training environment. This was supported by another’s suggestion to introduce elements like “time pressure and multi-tasking” to reflect clinical realities better.

The potential impact of VR on learning outcomes is multifaceted, with participants highlighting its capacity to enhance understanding and its limitations. One noted the psychological benefits, saying VR “could maybe make them a bit more confident,” suggesting VR's role in reducing anxiety around clinical settings.

### User-friendliness

Several participants highlighted the laborious setup process: “it can be too technical, and [students] have to familiarize themselves with it first.” One participant critiqued the VR software’s user interface, noting the difficulty in accessing essential features due to poorly designed menus: “it’s little user-friendly, frankly, it's quite poor.” “You spend so much time on the technical…”, another participant indicated the disproportionate focus on technical troubleshooting over educational content, revealing a misalignment of priorities where technological hurdles overshadow the learning objectives.

Several participants emphasized the necessity of familiarity with the system for meaningful use: “you spend so much time logging in, learning to move… it has to be more motivating.” This sentiment was mirrored by another who suggested a tutorial for smoother navigation: “Just getting to know the game…that button does this,” advocating for a user-friendly introduction to VR’s functionalities. The learning curve appeared steep but promised quicker adaptation with continued use, as another notes, “Once you've learned it, it will be faster next time.”

Prior experience with gaming and VR significantly influenced the perceived ease of adaptation. Several participants acknowledged the advantage of previous experience, while others, despite being seasoned users, expressed dissatisfaction with the time-consuming setup process. One appreciated the simplicity for beginners, “As a beginner, it was nice not to worry about, or work with, multiple factors,” suggesting that a clutter-free introduction aids learning. The diversity in backgrounds, as pointed out by participants, emphasized the need for tutorials catering to a broad range of users, from complete novices to those with extensive gaming or VR experience.

### Motivation and future use

The potential for VR in educational settings could be significantly enhanced with improvements in accessibility and application. One participant suggested a shift towards more familiar gaming formats could increase VR's utility: “if it was more like a computer game, it could be more realistic”. Another reflected on the possibility of personal practice, “if I had the option, I would use it to become more comfortable with it, to practice…medication calculation.” This was further underscored by another, suggesting that converting VR to a platform accessible at home, like a video game, could aid in “getting the practice without everyone needing to be in a medicine room.”

Despite the acknowledged potential, several barriers may hinder VR's integration into educational curriculums. One participant identified a significant obstacle related to VR equipment’s accessibility: “not everyone has VR equipment at home, and I doubt people would come to school to practice it in their free time.” Another mentioned concerns about the comfort level with the technology in shared spaces. Echoing this sentiment, another skeptically asked, “what’s the point of VR, it just takes time… I think it’s innovation just for the sake of the college being able to show they're doing something innovative.” Furthermore, another believed that if not for the long setup times, “it would be used more,” while another criticized the inefficiency of current implementations as “an unnecessary use of time.”

## Discussion

This study set out to investigate how nursing students perceive and assess the use of VR technology in their education, and how these insights can be leveraged by educators and policymakers to advance and innovate nursing education.

Participants explicitly stated that the VR technology and its content, specifically the Virtual Medicine Room, did not align with their actual academic needs. This finding is consistent with Gupta, Wilcocks [27], who emphasize the importance of focusing on end-user requirements in the development of educational and training VR software. Gupta, Wilcocks [27] advocate for ongoing dialogue throughout the development process to better meet defined needs and identify optimal solutions. For our study, this misalignment suggests that VR applications in nursing education need to be more closely tailored to students’ specific learning objectives and practical requirements.

Our findings suggest that VR technology is primarily seen as a complement to traditional training methods rather than a replacement. This aligns with Barteit, Lanfermann [28], who highlight similar challenges associated with using VR in education. The challenges associated with the utility and relevance of this technology, according to the informants, also revolved around the time and resources related to the use of VR in education, as well as concerns that the focus and allocation of resources to VR might come at the expense of other forms of training, as also found by Alalwan, Cheng [29]. These concerns reflect the need for a balanced integration of VR, ensuring it supplements rather than detracts from other essential training methods.

The implementation of VR in this study mirrors more traditional approaches to medication management training, such as classroom teaching, e-learning, and simulated training in physical replicas of medication rooms. While these methods are well-established, the direct transposition of such practices into VR highlights an important critique: “Where is the pedagogy?” [30]. As Mikropoulos and Natsis observed, many VR studies lack a clear pedagogical model, often defaulting to implied constructivist approaches like experiential learning or problem-based learning [31]. One alternative is to incorporate gamification principles into medication management training. A more gamified approach—featuring interactive tasks, adaptive challenges, and real-time feedback—could better utilize VR’s affordances while enhancing engagement and skill development. For example, introducing time-based challenges or interactive feedback loops could simulate real-world pressures, fostering deeper learning through problem-solving, a core tenet of constructivist learning theory [32].

Realism was a recurring theme among informants. They commented on the difficulty of translating the complexity of the real world into a virtual setting, noting the absence of elements like time pressure and chaos. The findings suggest that limited realism may affect the perceived transferability and applicability of this type of virtual training to real-world situations. This issue has been discussed by Shorey and Ng [5] and Weber, Weibel [33], who also found that the perceived lack of realism can hinder the effectiveness of VR training. For our study, this underscores the importance of enhancing the fidelity of VR simulations to better prepare students for actual clinical environments. Notably, while some participants criticized the lack of realism, others suggested that the virtual medication room could benefit from a design inspired by computer games. This feedback raises an important distinction between *literal realism*—attempting to replicate clinical settings—and *perceived realism* achieved through intuitive and immersive design, as often seen in gamified environments [34]. By incorporating game-inspired design elements—such as intuitive navigation, feedback mechanisms, and interactive learning tasks—educational VR applications may enhance users' perceived realism and overall immersion [35]. This approach could bridge the gap between the complexity of real-world clinical practice and the limitations of current VR training, ultimately improving the training’s effectiveness and relevance.

A key finding in this study was the participants’ mixed experiences of *presence*, a defining feature of virtual reality [33]. Presence refers to the sense of “being there” in a virtual environment, which is essential for creating an immersive and effective learning experience [36]. Some participants reported strong feelings of presence, such as “it felt like I was actually in the medication room at a hospital,” indicating that the VR simulation successfully facilitated a sense of immersion. However, participants also described moments of a *break in presence*, particularly due to technical difficulties and a steep learning curve. Such breaks in presence are shown to diminish user engagement and reduce the overall effectiveness of VR applications [37]. These findings underscore the importance of balancing technical performance and usability in VR applications. To optimize presence, it is essential to streamline technical aspects, improve user interfaces, and reduce cognitive load [38]

Informants’ reflections on the potential learning outcomes of this type of training and how one might observe these effects suggest that quantifying outcomes can be challenging, as indicated by Pellas, Mystakidis [39]. The informants believed that primarily qualitative measurements should be used to observe whether virtual training can increase students’ sense of safety and self-confidence, as demonstrated in other studies [40, 41]. This indicates a preference for evaluating the impact of virtual training through qualitative rather than quantitative metrics, focusing on aspects such as perceived security and confidence enhancement. This preference stands in contrast to the identified need for further research focusing on quantitative and objectively measurable outcomes of using VR technology in education [17]. Our study thus highlights a gap in the current evaluative frameworks for VR in education, suggesting a dual approach that incorporates both qualitative and quantitative measures.

User-friendliness emerged as a key theme among the informants, with particular emphasis placed on technological challenges. Similar concerns are echoed in other studies [42, 43], which highlight how technological difficulties with VR technology significantly impact the overall experience. Time investment is central to the feedback from informants, especially regarding the initial time spent becoming acquainted with and understanding both the technology and software. The informants expressed that this diversion of time and focus away from the subject matter and learning process was a significant concern. These findings align with other research [44–46], which underscores similar challenges and particularly emphasizes cognitive load [47] as a crucial factor in understanding how this can lead to varying outcomes for individual learning through the use of VR technology in education. This indicates that the usability of VR systems must be enhanced to reduce cognitive load and facilitate smoother integration into the learning process.

The feedback from participants indicates that it is essential for the software to integrate a tutorial, which can provide guidance both initially and as needed throughout, on the functions of both the hardware and software. Research by Miguel-Alonso, Rodriguez-Garcia [48] demonstrates that users consider such guidance crucial for a positive experience using VR technology. Feedback from participants consistently indicated that head-mounted displays (HMDs) were perceived as user-unfriendly and inaccessible, whereas Desktop VR on a PC was deemed more suitable due to its familiarity and widespread availability. This format could facilitate training from home, a feature many participants desired. Research suggests there is little to no difference in learning outcomes between HMDs and Desktop VR. Contrarily, the use of HMDs might be counterproductive, as they can introduce technological challenges, cybersickness, and distractions from learning tasks [43, 49]. For our study, this preference for desktop VR suggests that future VR applications should consider user familiarity and accessibility to enhance effectiveness and adoption.

The participants’ motivation to adopt VR technology as part of their education was significantly influenced by their experiences of utility and relevance, as well as user-friendliness, pertaining to both the VR technology itself and the application of the Virtual Medicine Room. This is related to the connection between perceived usefulness, perceived ease of use, and intention to use, as described by Davis [20] in the technology acceptance model (TAM), and which continues to be supported in later works and developments of the model [9, 21–23]. Transferred to our research, this implies that how participants experienced utility and relevance, and user-friendliness, will impact their motivation for future use, which should also be focal points for the future development of both hardware and software in the use of VR technology in education. This linkage highlights the importance of designing VR applications that are not only technically advanced but also practically relevant and easy to use, to foster higher acceptance and sustained use.

Participants were skeptical about the future use of VR technology in their education as it is currently presented. Among the most prominent concerns were doubts regarding the realistic availability of VR technology for students. Additionally, they noted that VR technology as a pedagogical tool barely meets their actual needs. Direct feedback also included reflections on whether the primary motivation was more about the university’s need to demonstrate innovation rather than a genuine academic necessity. Research on factors influencing motivation for future use of VR technology suggests that negative experiences with software or applications can impact the assessment of VR technology’s utility in education [50]. Thus, these factors must be considered in a broader understanding of what influences the evaluation of a given technology. Our findings emphasize the need for a critical evaluation of the actual benefits versus the perceived novelty of VR, ensuring that its implementation is driven by genuine educational needs rather than institutional prestige.

Future research should prioritize understanding our interactions with new technologies, such as VR, to improve our ability to assess their suitability as educational tools. This deeper understanding will enhance our competence in evaluating whether technologies like VR are indeed the most effective means for achieving educational goals. In cases where VR or similar technologies are considered the most suitable approach, future research should aim to provide the knowledge needed to develop solutions that are firmly grounded in specific learning objectives and tailored to the needs of end-users.

### Limitations

The relatively small sample size and the specific context of nursing education may limit the generalizability of the findings. As such, the experiences and perceptions of our participants may not be representative of all nursing students or those in other disciplines. Second, the use of focus groups, while valuable for eliciting detailed discussions, may have influenced participants to conform to group dynamics, potentially leading to less diversity in individual opinions. Third, the VR technology and applications used in this study were limited to one type of software and setting—the Virtual Medicine Room—thus the findings might not apply to other VR educational tools or contexts.

## Conclusion

This study illuminated key insights into integrating VR technology into nursing education, highlighting student perceptions concerning its utility, relevance, and user-friendliness. Although students recognized the potential benefits of VR in enhancing educational outcomes, there were notable concerns regarding whether the VR technology and its content—specifically the Virtual Medicine Room—adequately met their real educational needs and objectives. Our findings emphasize the importance of closely aligning VR technologies with end-user requirements, suggesting a continuous dialogue throughout the development process.

## Data Availability

The research data supporting the results of this manuscript consist of transcriptions of focus group interviews. Due to privacy and confidentiality agreements with participants, these data are not publicly available. Researchers who wish to access the data for verification purposes can contact the corresponding author to discuss conditions for access.

## Abbreviations

HMD: Head-mounted displays
TAM: Technology acceptance model
VR: Virtual reality
